# Cervical spinal cord bullet fragment removal using a minimally invasive surgical approach: a case report

**DOI:** 10.1186/1752-1947-6-235

**Published:** 2012-08-09

**Authors:** Cort D Lawton, Zachary A Smith, Koichi Sugimoto, Justin S Smith, Richard G Fessler

**Affiliations:** 1 Department of Neurological Surgery, Northwestern University, 676 N. St. Clair Street, Suite 2210, Chicago, IL 60661, USA; 2 Neurological Surgery, UVA Spine Center, 415 Ray C. Hunt Dr., Charlottesville, VA 22903, USA

**Keywords:** Bullet, Gunshot wound, Intramedullary, Minimally invasive spine surgery, Spinal cord

## Abstract

**Introduction:**

We present a case of penetrating gunshot injury to the high-cervical spinal cord and describe a minimally invasive approach used for removal of the bullet fragment. We present this report to demonstrate technical feasibility of a minimally invasive approach to projectile removal.

**Case presentation:**

An 18-year-old African-American man presented to our hospital with a penetrating gunshot injury to the high-cervical spine. The bullet lodged in the spinal cord at the C1 level and rendered our patient quadriplegic and dependent on a ventilator. For personal and forensic reasons, our patient and his family requested removal of the bullet fragment almost one year following the injury. Given the significant comorbidity associated with quadriplegia and ventilator dependency, a minimally invasive approach was used to limit the peri-operative complication risk and expedite recovery. Using a minimally invasive expandable retractor system and the aid of a microscope, the posterior arch of C1 was removed, the dura was opened, and the bullet fragment was successfully removed from the spinal cord.

**Conclusions:**

Here we describe a minimally invasive procedure demonstrating the technical feasibility of removing an intramedullary foreign object from the high-cervical spine. We do not suggest that the availability of minimally invasive procedures should lower the threshold or expand the indications for the removal of bullet fragments in the spinal canal. Rather, our objective is to expand the indications for minimally invasive procedures in an effort to reduce the morbidity and mortality associated with spinal procedures. In addition, this report may help to highlight the feasibility of this approach.

## Introduction

Gunshot injury to the spine accounts for 13% to 17% of all spinal cord injuries annually [1]. Spinal cord decompression and bullet removal remain controversial and can be associated with a high rate of complications [1-5]. Minimally invasive (MI) approaches have been applied to multiple spinal pathologies, including degenerative, traumatic, and neoplastic, in order to decrease morbidity and expedite recovery [6-13]. We present the case of a ventilator-dependent quadriplegic patient, who presented 11 months following a gunshot wound, requesting removal of the bullet fragment from the high-cervical spinal cord. We describe the use of a muscle splitting, MI approach to successfully remove the fragment from the spinal cord at the C1 level.

## Case presentation

**Figure 1:**
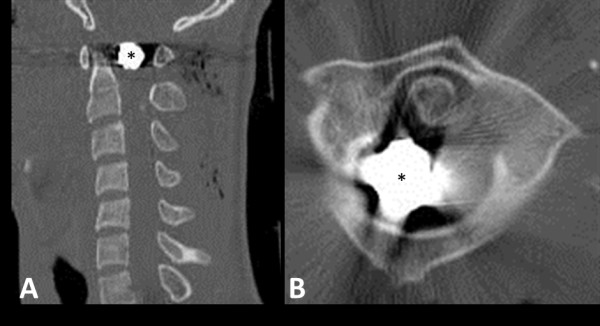
**Radiographic imaging of a retained bullet fragment in the high-cervical spine.** Sagittal (**A**) and axial (**B**) computed tomography (CT) images show the relationship of the fragment (designated by *) to the arch of C1 and the dens of C2. Note the fracture of the right posterior arch of C1 (arrow).

A previously healthy 18-year-old African-American man suffered a high-cervical gunshot wound to the neck while driving. This injury resulted in quadriplegia at the C2 sensory level and ventilator dependence. Imaging studies demonstrated a retained bullet fragment in the spinal canal at the level of C1 (Figure 1A,B). His initial care was received at another medical center where he was evaluated. No surgical intervention for the gunshot wound was pursued. He was ultimately discharged to a rehabilitation center after a tracheostomy was performed and a gastrostomy tube and inferior vena cava (IVC) filter were placed. His recovery was complicated by cardiac arrest, with successful resuscitation, and pneumonia.

Our patient and his family presented to our clinic 11months after the injury seeking removal of the bullet fragment for personal and forensic reasons. He remained without neurologic improvement, and the family was clearly counseled that such a procedure would be extremely unlikely to result in any neurological recovery.

**Figure 2:**
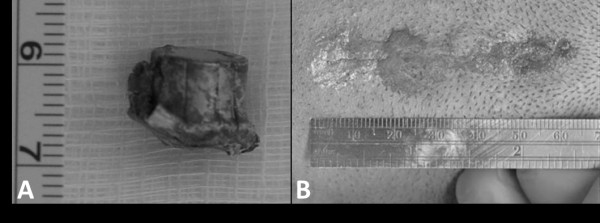
(A) Photograph of the extracted bullet fragment and (B) photograph of our patient’s neck in the prone position, demonstrating the post-operative skin incision and the site of bullet penetration.

After induction of general anesthesia, a Mayfield head frame was placed, and our patient was turned to a prone position on a standard operative table with a Wilson frame. The head frame was secured to the operative table, and the wound was prepared and draped in a sterile fashion. Fluoroscopy was used to determine the bullet location. An approximately 5cm midline longitudinal incision was created, centered over the level of the bullet fragment. Under fluoroscopic guidance, the METRx™ X-Tube Retraction System (Medtronic Sofamor Danek, Minneapolis, MN, USA) was placed, with sequential dilation of the muscle and exposure of the posterior arch of C1. The retractor was secured to the operative table and expanded to a working diameter of approximately 5cm. An operative microscope was then brought into the field for visualization. The posterior arch of C1 was removed using a Kerrison #2 bone punch, and the underlying dura was exposed. The dura was incised and found to be remarkably adherent to an underlying mass of granulation tissue. Rhoton™ microdissectors were used to carefully dissect the granulation tissue and expose the bullet fragment. The fragment was dissected free from the surrounding granulation tissue and removed (Figure 2A). The dura was closed using a running 5-0 prolene suture and a custom needle driver and knot pusher (Scanlan International, Saint Paul, MN, USA). The wound was closed using deep and subcutaneous absorbable interrupted sutures, and the skin surface was covered with surgical glue.

The surgical time was approximately two hours and 20 minutes, and the estimated blood loss was 50cm^3^. The procedure and hospital stay were uncomplicated. At a 6-month follow-up, our patient remained at his pre-operative neurologic baseline and free from evidence of complications with only a small scar from the site of skin incision and bullet penetration (Figure 2B).

## Discussion

There are few absolute indications for the removal of a bullet lodged in the spinal canal, including neurological deterioration, infection, and lead or copper toxicity [2]. Whether to perform such a procedure in situations outside of these indications remains controversial. We present a case where a quadriplegic, ventilator-dependent patient requested removal of a bullet fragment retained in the high-cervical spinal cord. Given the comorbidity generally associated with ventilator-dependent quadriplegia, exemplified by our patient’s own history of cardiac arrest and pneumonia, we sought to minimize the morbidity of the planned procedure. Further, it should be noted that many patients who present following a high-velocity penetrating trauma are commonly medically unstable. In these situations, a minimally invasive approach of this nature may be advantageous given that a larger, open operation may be medically contraindicated.

Standard open approaches for removal of bullet fragments from the spinal canal have been associated with a high rate of complications. Simpson *et al*. retrospectively reviewed 160 cases of penetrating spinal injury, including 142 cases of gunshot wounds and 18 stab wounds [14]. Complications included meningitis, cerebrospinal fluid leakage, and wound infections. The complication rate was considerably higher in the patients who were surgically treated (22%) compared with patients who were treated non-surgically (7%). Minimally invasive procedures to access the spine have been developed in order to reduce approach-related morbidity and/or post-operative complications [6,9,10]. Initial applications were primarily confined to discectomies and decompressions for degenerative spine disease, [6,8,10,11,15,16] but with recognition of the benefits and further advances in technology, the potential indications have markedly expanded [12,13,17-19]. The ability to safely and effectively access the spinal intradural compartment through a MI approach has been reported [7,12]. In addition, the removal of bullet fragments in the lumbar spine has also been previously discussed [20,21].

When contemplating removal of a retained bullet fragment in the spinal canal, it is important that the indications and expectations are clear. These injuries are often treated non-operatively, especially in cases of complete neurological deficit. However, in select circumstance, when surgery is elected, a minimally invasive approach may be both safe and feasible.

## Conclusions

Here, we have demonstrated the technical feasibility of removing an intramedullary foreign object from the high-cervical region. Although surgical indications remain controversial, removal of a foreign object, such as a bullet fragment, from the high-cervical spinal cord may be safely and effectively achieved through a MI approach.

## Consent

Written informed consent was obtained from the patient for publication of this manuscript and any accompanying images. A copy of the written consent is available for review by the Editor-in-Chief of this journal.

## Competing interests

Zachary A. Smith’s contributions were supported by the CNS Spine Fellowship Award and the AANS/CNS Spine Section Apfelbaum Grants.

## Authors’ contributions

CDL was a major contributor in writing the manuscript, provided critical revisions, and created the figures. ZAS provided critical revisions and contributed to the writing, concept, design, revision, and completion of the manuscript. KS was a significant contributor to the writing of this manuscript. JSS was a contributor in writing the manuscript, the conception, and the design. RGF provided critical revisions and gave the final approval of the version to be published. All the contributing authors have read and approved the final manuscript.
